# Co-expression of SOX2 and HR-HPV RISH predicts poor prognosis in small cell neuroendocrine carcinoma of the uterine cervix

**DOI:** 10.1186/s12885-021-08059-1

**Published:** 2021-03-31

**Authors:** Shi-Wen Zhang, Rong-Zhen Luo, Xiao-Ying Sun, Xia Yang, Hai-Xia Yang, Si-Ping Xiong, Li-Li Liu

**Affiliations:** 1 Department of Pathology, the Eighth Affiliated Hospital, Sun Yat-sen University, Shenzhen, 51800 China; 2 Department of Pathology, Sun Yat-sen University Cancer Center, Guangzhou, 510060 China; 3 Department of Gynecological Oncology, Sun Yat-sen University Cancer Center, Guangzhou, 510060 China

**Keywords:** SOX2, HR-HPV RISH, P16^INK4A^, SCNEC, Prognosis

## Abstract

**Background:**

Small cell neuroendocrine carcinoma of the uterine cervix (SCNEC) is a rare cancer involving the human papilloma virus (HPV), and has few available treatments. The present work aimed to assess the feasibility of SOX2 and HPV statuses as predictive indicators of SCNEC prognosis.

**Methods:**

The associations of SOX2 and/or high-risk (HR)-HPV RNA in situ hybridization (RISH) levels with clinicopathological characteristics and prognostic outcomes for 88 neuroendocrine carcinoma (NEC) cases were analyzed.

**Results:**

Among these patients with SCNEC, SOX2, P16^INK4A^ and HR-HPV RISH expression and SOX2/HR-HPV RISH co-expression were detected in 68(77.3%), 76(86.4%), 73(83.0%), and 48(54.5%), respectively. SOX2-positive and HR-HPV RISH-positive SCNEC cases were associated with poorer overall survival (OS, *P* = 0.0170, *P* = 0.0451) and disease-free survival (DFS, *P* = 0.0334, *P* = 0.0309) compared with those expressing low SOX2 and negative HR-HPV RISH. Alternatively, univariate analysis revealed that SOX2 and HR-HPV RISH expression, either separately or in combination, predicted the poor prognosis of SCNEC patients. Multivariate analysis revealed that the co-expression of SOX2 with HR-HPV RISH may be an independent factor of OS [hazard ratio = 3.597; 95% confidence interval (CI): 1.085–11.928; *P* = 0.036] and DFS [hazard ratio = 2.880; 95% CI: 1.199–6.919; *P* = 0.018] prediction in SCNEC.

**Conclusions:**

Overall, the results of the present study suggest that the co-expression of SOX2 with HR-HPV RISH in SCNEC may represent a specific subgroup exhibiting remarkably poorer prognostic outcomes compared with the expression of any one marker alone.

**Supplementary Information:**

The online version contains supplementary material available at 10.1186/s12885-021-08059-1.

## Introduction

Small cell neuroendocrine carcinoma of the uterine cervix (SCNEC) is a highly aggressive and rare malignant cervical cancer (< 3%). The incidence of lymph node and distant metastases is high in the early stage of SCNEC, although SCNEC is usually only detected at the advanced stage [1–3]. SCNEC has a poor prognosis, which is closely related to the stage at diagnosis. Particularly, the 5-year survival rate for early SCNEC cases is 30–46%, while it is only 0–15% for patients at the advanced stage [4]. Despite the increase in multidisciplinary therapies, patients with advanced SCNEC still have a poor prognosis [5]. Therefore, it is important to improve SCNEC prognosis.

The sex-determining region Y-box 2 (*SOX2*) gene, located on chromosome 3q26.3-q27, belongs to the SOX family [6]. Notably, SOX2 has been recognized as a potent transcription factor involved in self-renewal, maintenance of stem cell properties, and pluripotency in embryonic stem cells [6, 7]. SOX2 plays a vital role in tumor development, progression, and cell survival in various cancer types [8–10]. A few studies have reported that SOX2 is overexpressed in cervical squamous cell carcinoma (SCC), and plays an important role in the progression from squamous dysplasia to SCC. The expression of SOX2 is correlated with the degree of differentiation of SCC, and up-regulation of SOX2 has been shown to enhance cervical cancer cell invasion and migration in vitro [11, 12]. The small infiltrating cancer nests surrounding CIN 3 margins or the CIN 3-like SCC with deep invasion generally display a decreased SOX2 level locally, and this indicates reduced SOX2 expression during invasive growth [13]. SOX2 has been reported to be related to HPV infection in previous studies [14, 15]. However, its expression, clinical significance and the association between SOX2 and HPV status in SCNEC have not been evaluated.

Numerous studies have demonstrated a closed etiopathogenetic relationship between the development of cervical cancers and high-risk (HR) human papilloma virus (HPV) infection [16]. The occurrence of SCC and adenocarcinoma is associated with HPV16 infection, while that of SCNEC is associated with HPV18 infection [17]. HPV infection is detected using a variety of approaches, such as polymerase chain reaction (PCR), immunohistochemistry (IHC), and in situ hybridization (ISH) [18, 19]. Previous studies have shown that HPV mRNA detection and P16^INK4A^/Ki67 IHC are valuable biomarkers for HPV oncogenic expression [20]. The detection of mRNA expression indicates changes at the molecular level, and mRNA amplification becomes a poor prognostic factor when persistently infected with a highly oncogenic type, such as HPV 18 [21]. Furthermore, HPV mRNA expression, detected using HR-HPV RISH, in SCNEC has not been investigated extensively.

The present study retrospectively examined the expression levels of SOX2 and HPV mRNA in SCNEC and investigated the relationships between the expression levels and clinicopathological characteristics in SCNEC cases.

## Materials and methods

### Patients and samples

In the present retrospective study, we enrolled 88 patients with histologically confirmed SCNEC, who had under surgical resection at Sun Yat-sen University Cancer Center between January 2010 and December 2014. Patients were enrolled when they were diagnosed with primary SCNEC, with available clinical information. The last follow-up was conducted in June 2020. The study protocol was approved by the Institutional Ethical Board of Sun Yat-sen University Cancer Center. The raw data relevant to the study were imported into the Research Data Deposit public platform (www.researchdata.org.cn; RDD approval number: RDDA2020001710).

#### Tissue microarray (TMA) establishment and IHC

SCNEC tumor tissues and lymph node metastatic tissues, were obtained to construct a TMA. Thereafter, the constructed TMA block was sectioned (thickness, 4 μm) and stained for immunohistochemical analyses. Sections were de-waxed in xylene and ethanol and treated with 3% hydrogen peroxide diluted with methanol. Thereafter, avidin-biotin was used to block all sections overnight at 4 °C. The sections were then incubated with anti-SOX2 (ab134154, Abcam), anti-P16^INK4A^, anti-Synaptophysin (GT206529), anti-Chromogranin A (ZA-0507, Zhongshan, China), anti-CD56 (ZM-0057, Zhongshan, China), anti-MSH6 (SP93) (Roche, Germany), anti-MLH1 (Roche (M1), Germany), anti-PMS2 (Dako (EP51), Germany), anti-Ki-67 (ZA0502), and anti-MSH2 (ZA0622, Zhongshan, China) antibodies. Phosphate buffer was used to wash the sections three times before incubation with biotinylated goat anti-mouse antibodies. Next, DAKO liquid 3, 3′-diaminobenzidine tetrahydro-chloride (DAB) was used for staining, and Mayer’s hematoxylin was used for counter-staining. The block-like and diffuse staining at every core was positive for P16^INK4A^, whereas the patchy or non-stained sections indicated a negative result. Additionally, ≥1% positively-stained cancer cell nuclei indicated a positive result for MSH2/MSH6/PMS2/MLH1. The SOX2 positive samples were scored as follows: 0, < 5% cells with positive staining; 1, 5–24% cells with positive staining; 2, 25–49% cells with positive staining; 3, 50–74% cells with positive staining; and 4, 75–100% cells with positive staining. Positive staining intensity was graded as follows: 0, 1, 2, and 3 suggested negative, weak, moderate, and strong staining, respectively. Then, the percentage score was multiplied by the intensity score to obtain the eventual score. Thereafter, 6.5 was used as the median IHC score to classify high or low SOX2 expression levels.

#### HPV subtypes

According to a previous study, the tumor tissues used in PCR should not be the same as those used in TMA [22]. HPV was detected using the Roche Cobas 4800 system (Pleasanton, CA), and the following 14 subtypes of HPV DNA were detected: 16, 18, 31, 33, 35, 39, 45, 51, 52, 56, 58, 59, 66, and 68.

### HPV E6/E7 mRNA in situ hybridization

The RNAscope scoring system was used to examine each specimen, as described in previous studies [23, 24]. HPV fluorescent in situ hybridization was performed using a chromogen and the RNAscope system (Advanced Cell Diagnostics; catalog no. 312598). The RNAscope probe HPV HR18 contains probes that target E6 and E7 mRNA in these high-risk subtypes: HPV16, 18, 26, 31, 33, 35, 39, 45, 51, 52, 53, 56, 58, 59, 66, 68, 73, and 82. The RNAscope results were classified into five degrees based on the following scoring guidelines: 0, no staining or <  1 dot in 10 cells (40x); 1, 1–3 dots in each cell (20–40x); 2, 4–10 dots in each cell as well as a few dot clusters (20–40x); 3, > 10 dots in each cell as well as dot clusters in < 10% positive cells (20x); 4, > 10 dots in each cell as well as dot clusters in > 10% positive cells (20x). An RNAscope score ≥ 1 indicated positivity.

#### Statistical analysis

The SPSS 19.0 software was used for data analyses. The expression of SOX2 in different SCNEC subgroups was compared using an unpaired T-test. Correlations of SOX2 and P16^INK4A^ with the HPV mRNA expression levels and clinicopathological parameters in patients with SCNEC were analyzed using the chi-square test. Kaplan-Meier analysis was used to analyze overall survival (OS) and disease-free survival (DFS), while the log-rank test was used for comparison. The correlation of prognosis was analyzed using univariate and multivariate Cox regression analyses. *P* < 0.05 (two-sided) indicated statistical significance. To construct a nomogram, the predictive power of each variable for OS and DFS was evaluated using univariate Cox regression. Thereafter, the significant variables were used in multivariate Cox analysis. To determine independent prognostic variables, the Akaike information criterion (AIC) score was used for backward selection for suitable variables. Finally, the variables were enrolled for the nomogram construction, with 1-, 3-, and 5-year OS and DFS selected as the primary endpoints. To evaluate the nomogram predicting power, a concordance index (C-index) with receiver operating characteristic (ROC) curve analysis was applied [25]. The discrimination of the predicted values from the actual values was visualized by generating calibration curves for 1- and 3-year OS data [26].

## Results

### Patient characteristics

The characteristics of 88 patients with SCNEC are summarized in Table S1. Sixty-six patients (SCNEC-alone, 75%) presented with only small cell carcinoma components, and Twenty-two patients (SCNEC-mix, 25%) presented with small cell carcinoma mixed with other epithelium-derived tumors (SCC, adenocarcinoma, and others) were noted. There were 79 (89.8%) and 9 (10.2%) cases at FIGO stages I–IIA and IIB–IV, respectively, and a median 30.6-month follow-up was conducted to examine OS and DFS.

### Expression of SOX2 and P16^INK4A^ in SCNEC tissues was detected using IHC

**Fig. 1:**
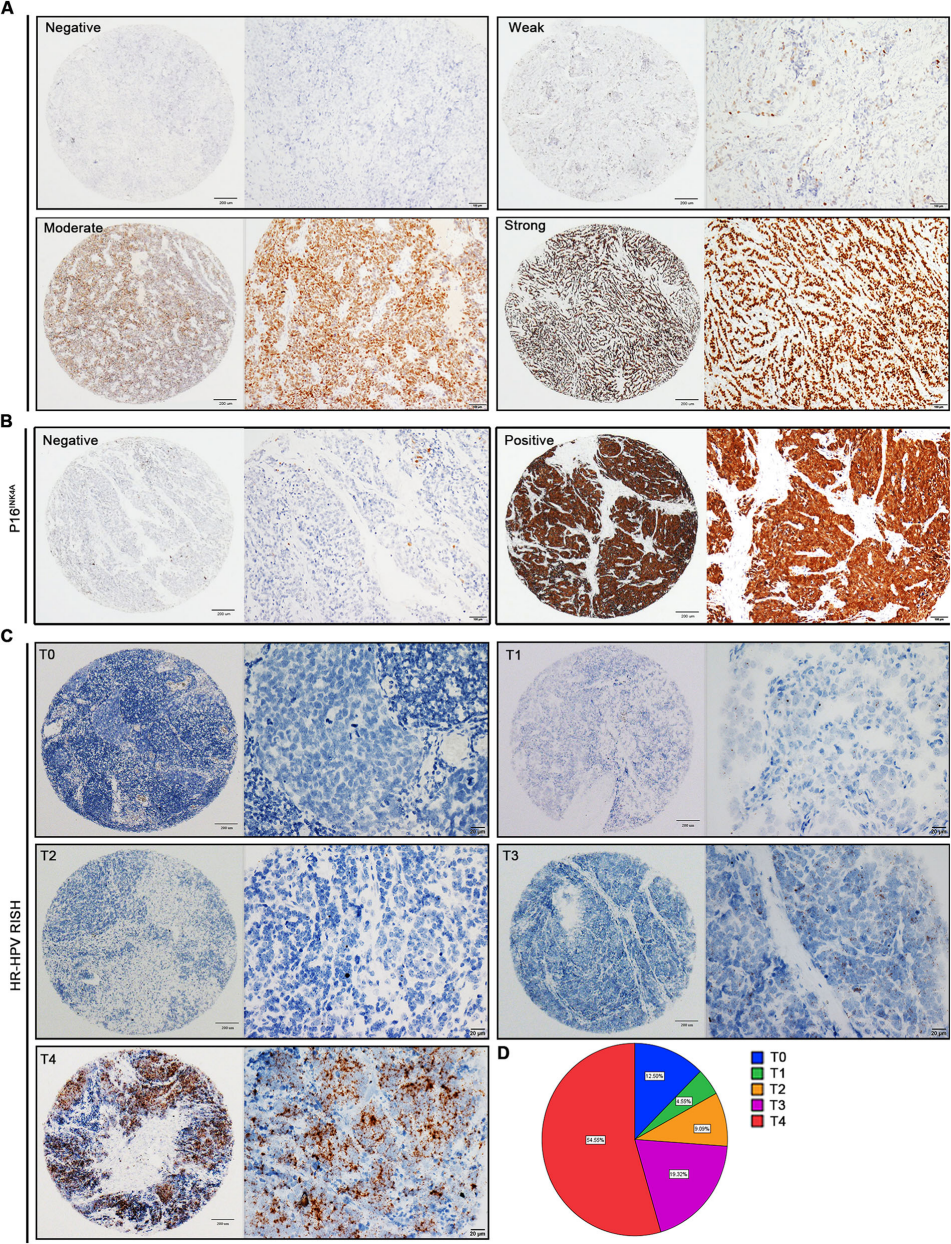
Expression of SOX2, P16^INK4A^, and HR-HPV RISH in SCNEC. **a** Immunohistochemical staining showing the SOX2 level in tissue microarray (TMA), with negative, weak, moderate, and strong intensity staining. **b** Negative and positive immunohistochemical staining for P16^INK4A^ expression in tumor tissues. **c** HPV mRNA was detected using HR-HPV RISH in tumor tissues (T). Representative images of scores 0 (T0), 1 (T1), 2 (T2), 3 (T3), and 4 (T4) are shown. **d** The proportion of HR-HPV RISH scores in SCNEC tissues

To confirm the SOX2 and P16^INK4A^ expression profiles in SCNEC, a total of 88 paraffin-embedded SCNEC tissue samples, with the corresponding clinicopathological data, were harvested to construct the TMA cohort. SOX2 was primarily located in the nucleus, while P16^INK4A^ was observed in the cytoplasm (Fig. 1a and b). SOX2 expression levels within tumor tissues were observed and classified as negative, weak, moderate, or strong staining (Fig. 1a). Among these patients with SCNEC, SOX2, P16^INK4A^, and HR-HPV RISH were detected in 68(77.3%), 76(86.4%), and 73(83.0%), respectively (Fig. S1A). Furthermore, the positive expression rates of SOX2 in the SCNEC-alone and SCNEC-mix groups were 72.7% (48/66) and 90.9% (20/22), respectively. SOX2 expression was not significantly different between groups (*P* = 0.1660) (Fig. S1B).

### Expression of HPV mRNA in SCNEC was detected using the RNAscope technique

In situ expression of HPV mRNA was detected using RNAscope and scored accordingly. HPV mRNA was detected predominantly in the cytoplasm of cancer cells with variable staining intensity. The scores of 0 to 4 and the proportions of T1–4 are shown in Fig. 1c and d. The HPV mRNA-positive rate was 86.4% (76/88) in patients with SCNEC, and the expression rates in SCNEC-alone and SCNEC-mix were 81.8% (54/66) and 100% (22/22), respectively (Fig. S1A and S1C). T-test analyses indicated (*P* = 0.2118) no significant difference between the groups (Fig. S1C). Another TMA contained 37 SCNEC cases with lymph node metastases. There was no significant difference in the HPV mRNA expression between primary tumor and lymph node metastases (*n* = 37; *P* = 0.1134) (Fig. S1D and S1E). Furthermore, the proportions of the combinations of HPV tests, SOX2- IHC and P16^INK4A^ expression levels are shown in Fig. S1F-I and Fig. S2.

### Relationship between SOX2, P16^INK4A^, and HPV mRNA expression and clinicopathological features in SCNEC

The association of SOX2, P16^INK4A^, and HPV mRNA expression with the clinicopathological characteristics in SCNEC is summarized in Table 1. SOX2 expression within tumors was significantly related to vascular invasion (*P* = 0.023) and relapse (P = 0.023), whereas SOX2 expression showed no significant correlation with other clinicopathological features of patients with SCNEC. However, P16^INK4A^ expression was only significantly related to Ki67 (*P* = 0.005). Notably, HPV mRNA levels were significantly correlated with FIGO staging (*P* = 0.021), pre-operative chemotherapy (*P* = 0.007), relapse (*P* = 0.027), neuroendocrine markers (P = 0.021), and pathological classification (*P* = 0.014). These data suggest that the overexpression of SOX2 and HPV mRNA potentially facilitates tumorigenesis and development of SCNEC.

**Table 1:** Patient demographics and clinical characteristics

	SOX2				P16^INK4A^				HR-HPV RISH			
Characteristic	n	Low	High	*P*	n	Neg.	Pos.	*P*	n	Neg.	Pos.	*P*
**Age (years)**				0.392				0.364				0.501
< 44	40	16	24		40	4	36		40	8	32	
≥ 44	48	15	33		48	8	40		48	7	41	
**HPV DNA (pg/ml)**				0.983				0.514				**0.028**
< 1	3	1	2		3	0	3		3	2	1	
≥ 1	56	19	37		56	7	49		56	9	47	
**FIGO stage**				0.178				0.816				**0.021**
≤ IIA	79	26	53		79	11	68		79	11	68	
≥ IIB	9	5	4		9	1	8		9	4	5	
**Pre-operative Chemotherapy**				0.084				0.681				**0.007**
No	56	16	40		56	7	49		56	5	51	
Yes	32	15	17		32	5	27		32	10	22	
**Post-operative Chemotherapy**				0.661				0.416				0.353
No	4	1	3		4	0	4		4	0	4	
Yes	84	30	54		84	12	72		84	15	69	
**Post-operative Radiotherapy**				0.120				0.109				0.826
No	33	15	18		33	2	31		33	6	27	
Yes	55	16	39		55	10	45		55	9	46	
**Pathological type**				0.699				0.473				**0.014**
SCNEC -alone	66	24	42		66	10	56		66	15	51	
SCNEC -mix	22	7	15		22	2	20		22	0	22	
**Tumor size (cm)**				0.358				0.112				0.896
< 2	11	6	5		11	3	8		11	2	9	
2–4	40	13	27		40	7	33		40	6	34	
≥4	37	12	25		37	2	35		37	7	30	
**Stromal invasion**				0.948				0.225				0.281
< 1/2	28	10	18		28	2	26		28	3	25	
≥1/2	60	21	39		60	10	50		60	12	48	
**Endometrial invasion**				0.178				0.816				0.170
No	79	26	53		79	11	68		79	12	67	
Yes	9	5	4		9	1	8		9	3	6	
**Parametrium invasion**				0.737				0.182				0.792
No	78	27	51		78	12	66		78	13	65	
Yes	10	4	6		10	0	10		10	2	8	
**CIN**				0.866				0.970				0.241
No	73	26	47		73	10	63		73	14	59	
Yes	15	5	10		15	2	13		15	1	14	
**LNM**				0.293				0.566				0.283
No	52	16	36		52	8	44		52	7	45	
Yes	36	15	21		36	4	32		36	8	28	
**Nerve invasion**				0.835				0.921				0.107
No	67	24	43		67	9	58		67	9	58	
Yes	21	7	14		21	3	15		21	6	15	
**LVI**				**0.023**				0.176				0.973
No	29	15	14		29	6	23		29	5	24	
Yes	59	16	43		59	6	53		59	10	49	
**Relapse**				**0.023**				0.297				**0.027**
No	54	24	30		54	9	45		54	13	41	
Yes	34	7	27		34	3	31		34	2	32	
**N-marker**				0.900				0.069				**0.021**
≤2 +	9	3	6		9	3	6		9	4	5	
> 2 +	79	28	51		79	9	70		79	11	68	
**MMR**				0.737				0.182				0.792
pMMR	78	27	51		78	12	66		78	13	65	
dMMR	10	4	6		10	0	10		10	2	8	
**Ki67(%)**				0.105				**0.005**				0.154
< 60	13	2	11		13	5	8		13	4	9	
≥60	75	29	46		75	7	68		75	11	64	

*CIN* Cervical Intraepithelial Neoplasia, *LNM* Lymph Node Metastasis, *LVI* Lymphatic Vessel Invasion, *N-marker* Neuroendocrine-marker, *MMR* Mismatch Repair

### SOX2 and HPV mRNA influenced the prognosis of patients with SCNEC

**Fig. 2:**
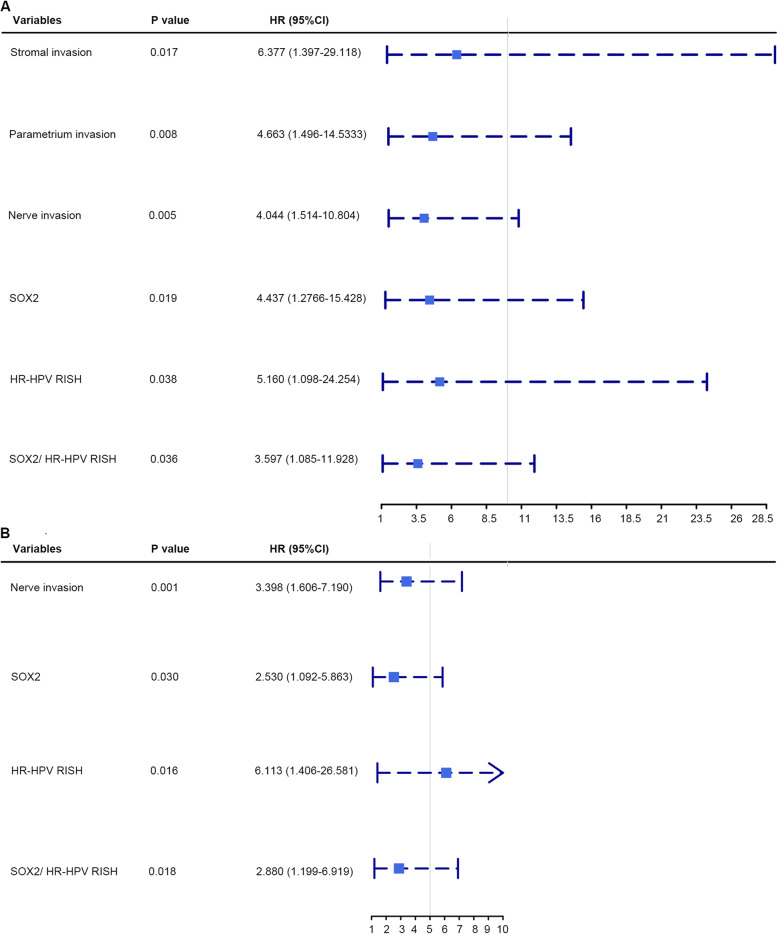
Forest plot displaying the hazard ratio and corresponding 95% confidence interval (CI) for overall survival (OS) and disease-free survival (DFS), based on Cox proportional hazards regression. **a** Forest plot showed the hazard ratio and 95% confidence interval for OS. **b** Forest plot showed the hazard ratio and 95% confidence interval for DFS

**Fig. 3:**
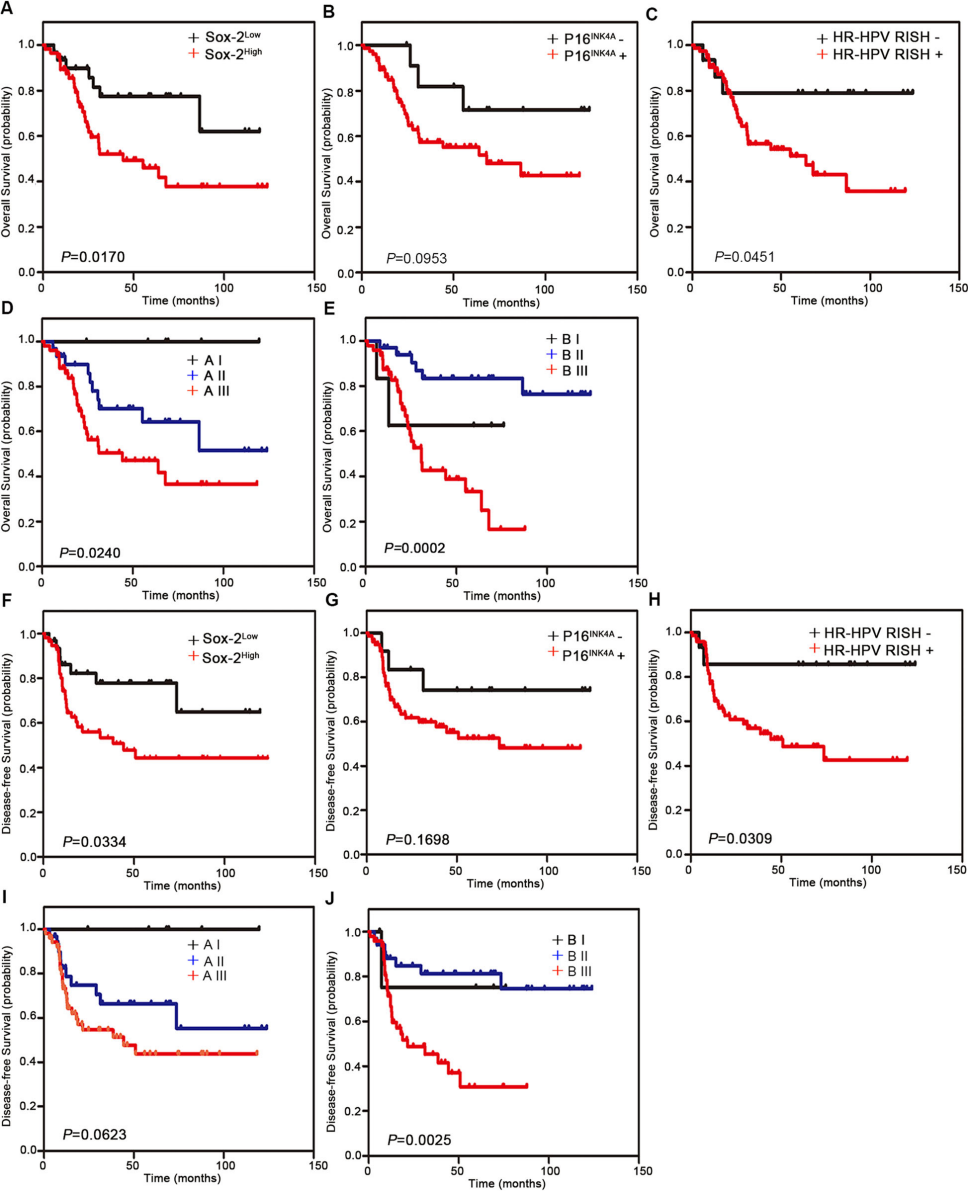
Kaplan-Meier survival curves for OS and DFS of small cell neuroendocrine carcinoma of the uterine cervix (SCNEC) cases according to SOX2, P16^INK4A^, and high-risk-human papilloma virus (HR-HPV) RISH expression. **a**–**c** OS according to SOX2, P16^INK4A^, and HR-HPV RISH expression status in patients with SCNEC. **d** OS according to a combination of SOX2/P16^INK4A^ co-expression. Group AI: SOX2^Low^/P16^INK4A^-; Group AII: SOX2^High^/P16^INK4A^- or SOX2^Low^/P16^INK4A^+; Group AIII: SOX2^High^/P16^INK4A^+. **e** OS according to a combination of SOX2- IHC/HR-HPV RISH co-expression. Group BI: SOX2^Low^/HR-HPV RISH-; Group BII: SOX2^High^/HR-HPV RISH- or SOX2^Low^/HR-HPV RISH+; Group BIII: SOX2^High^/HR-HPV RISH+. **f**–**h** DFS according to SOX2, P16^INK4A^, and HR-HPV RISH expression status in patients with SCNEC. **i** DFS according to a combination of SOX2/P16^INK4A^ co-expression. Group AI: SOX2^Low^/P16^INK4A^-; Group AII: SOX2^High^/P16^INK4A^- or SOX2^Low^/P16^INK4A^+; Group AIII: SOX2^High^/P16^INK4A^+. **j** DFS according to a combination of SOX2- IHC/HR-HPV RISH co-expression. Group BI: SOX2^Low^/HR-HPV RISH-; Group BII: SOX2^High^/HR-HPV RISH- or SOX2^Low^/HR-HPV RISH+; Group BIII: SOX2^High^/HR-HPV RISH+

**Fig. 4:**
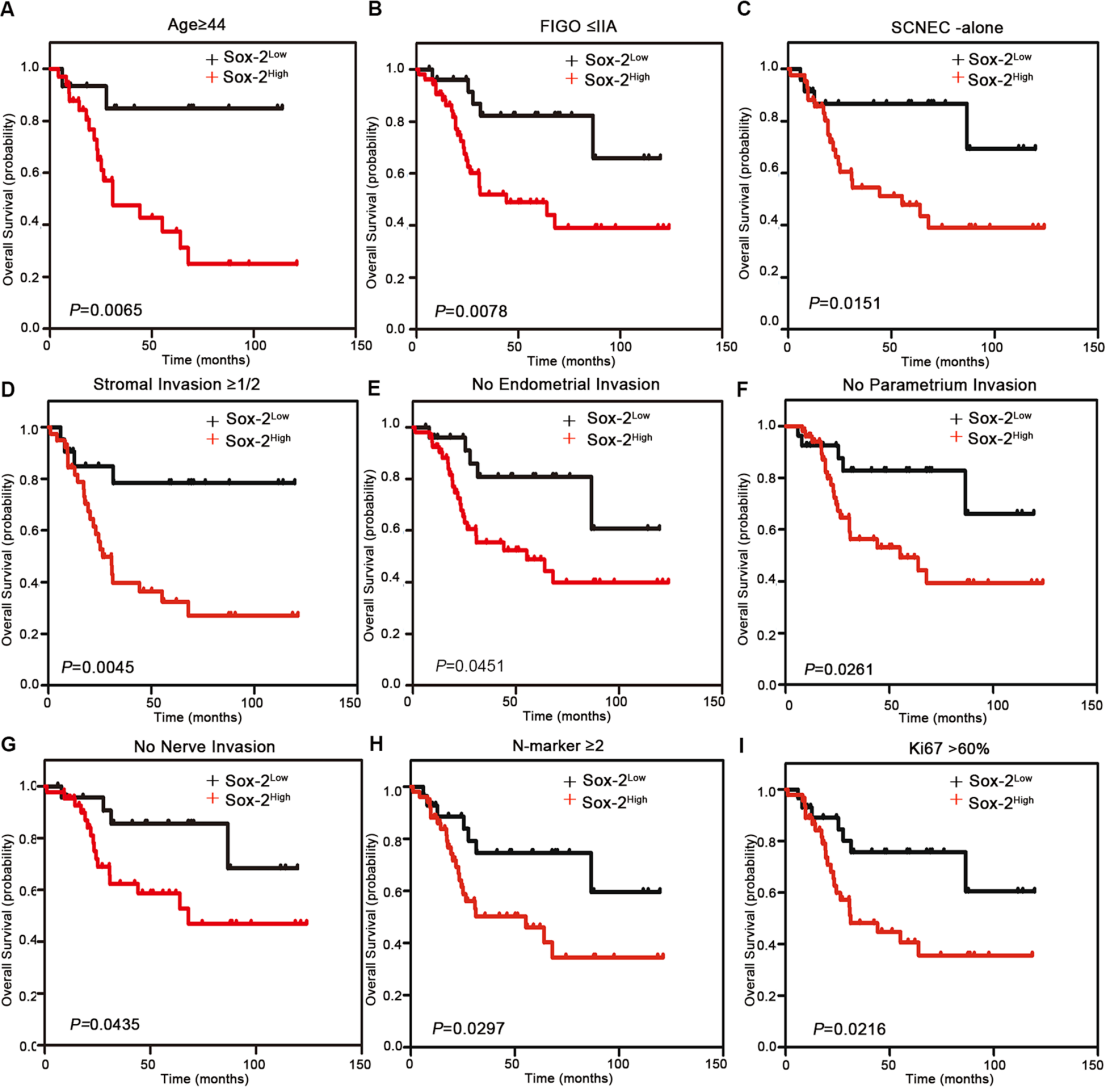
Stratified analysis of the OS-related SOX2 level. **a**-**i** Relationship of the SOX2 level with OS in specific groups

**Fig. 5:**
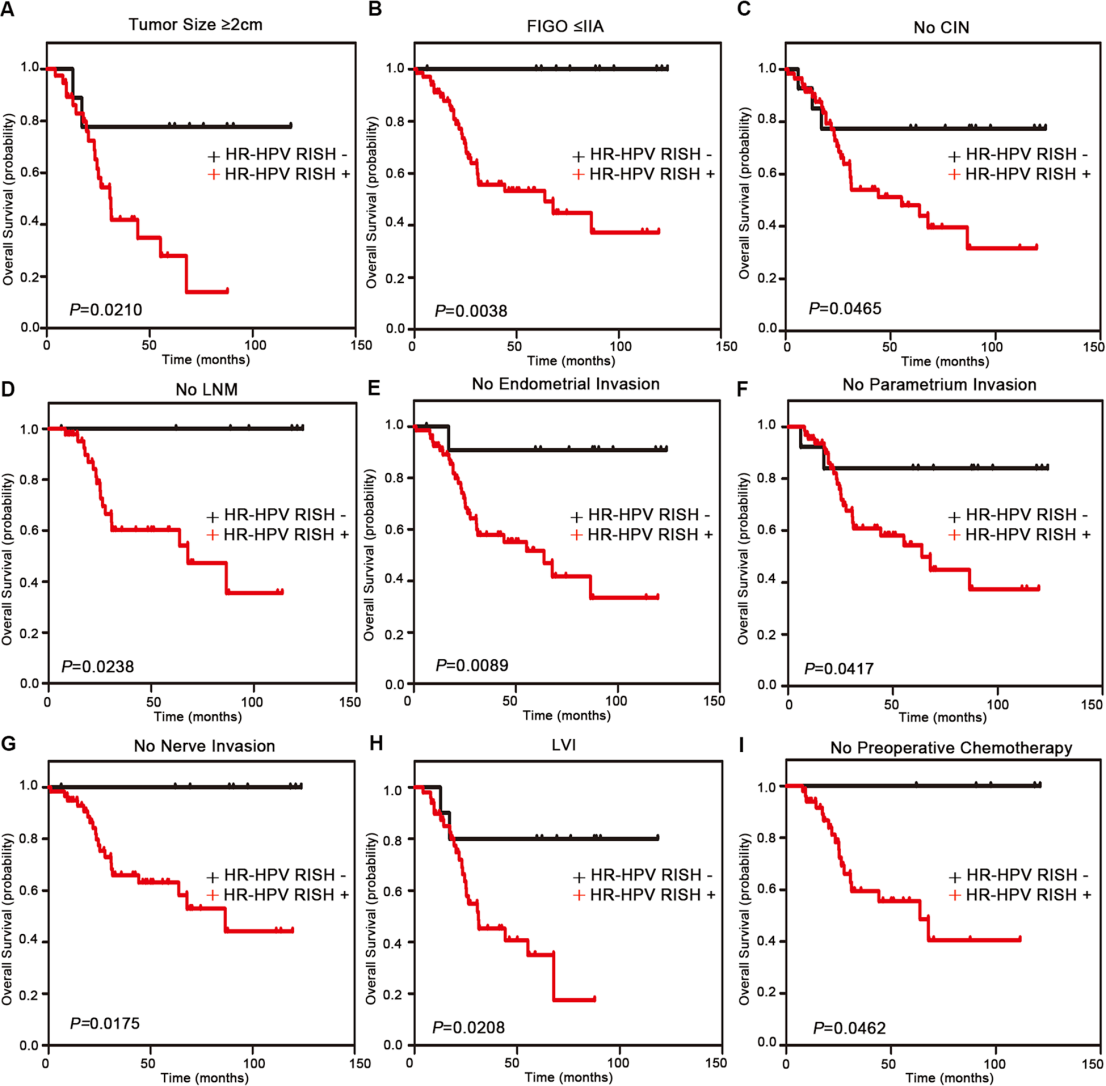
Stratified analysis of the OS-related HR-HPV RISH level. **a**-**i** Relationship of the HR-HPV RISH level with OS in specific groups

Tables 2 and 3 present the univariate and multivariate Cox hazards regression results, respectively. Univariate regression analysis suggested that HPV DNA, stromal invasion, parametrium invasion, nerve invasion, SOX2, HR-HPV RISH, SOX2/P16^INK4A^, and SOX2/HR-HPV RISH were related to patient OS (*P* < 0.05). Moreover, multivariate Cox regression showed that parametrium invasion (hazard ratio = 4.663; 95% CI: 1.496–14.533; *P* = 0.008), stromal invasion (hazard ratio = 6.377; 95% CI: 1.397–29.118; *P* = 0.017), nerve invasion (hazard ratio = 4.044; 95% CI: 1.514–10.804; *P* = 0.005), SOX2 (hazard ratio = 4.437; 95% CI: 1.276–15.428; *P* = 0.019), HR-HPV RISH (hazard ratio = 5.160; 95% CI: 1.098–24.254; *P* = 0.038), and SOX2 /HR-HPV RISH (hazard ratio = 3.597; 95% CI: 1.085–11.928; *P* = 0.036) may independently predict prognosis in SCNEC cases. Univariate regression analysis revealed that nerve invasion, SOX2, HR-HPV RISH, SOX2/P16^INK4A^, and SOX2/HR-HPV RISH were related to DFS (*P* < 0.05). Upon multivariate regression, it was observed that nerve invasion (hazard ratio = 3.398; 95% CI: 1.606–7.190; *P* = 0.001), SOX2 (hazard ratio = 2.530; 95% CI: 1.092–5.863; *P* = 0.030), HR-HPV RISH (hazard ratio = 6.113; 95% CI: 1.406–26.581; *P* = 0.016), and SOX2/HR-HPV RISH (hazard ratio = 2.880; 95% CI: 1.199–6.919; *P* = 0.018) were evidently related to DFS. Additionally, in this study, we created a forest plot to display hazard ratio together with the corresponding 95% CIs of OS and DFS, based on Cox proportional hazards regression (Fig. 2). In conformance to the above results, Kaplan-Meier curves for OS and DFS based on SOX2, P16^INK4A^, HR-HPV RISH, SOX2/P16^INK4A^, and SOX2 /HR-HPV RISH expression showed significant differences, which were verified through log-rank tests (Fig. 3). Survival analysis was also conducted, which revealed that SOX2 may be adopted to predict prognosis. Moreover, the statistical analyses indicated that SOX2 and HPV mRNA expression were associated with a series of pathological parameters related to OS (Figs. 4 and 5). Therefore, our data further suggest that SOX2, alone and in combination with HPV mRNA, is an independent prognostic marker for patients with SCNEC.

**Table 2:** Univariate and multivariate Cox proportional hazards regression analysis for OS

Variables	Univariate		Multivariate	
	HR (95% CI)	***P*** value	HR (95% CI)	***P*** value
Age (years) (< 44 vs. ≥44)	1.253 (0.632–2.482)	0.518		
HPV DNA (pg/ml) (< 1 vs. ≥1)	**0.240 (0.071–0.813)**	**0.022**		
FIGO stage (≤IIA vs. ≥IIB)	2.373 (0.914–6.158)	0.076		
Pathological type (SCNEC -alone vs. SCNEC -mix)	1.448 (0.661–3.174)	0.355		
Pre-operative Chemotherapy (No vs. Yes)	1.148 (0.721–2.787)	0.312		
Post-operative Chemotherapy (No vs. Yes)	0.514 (0.122–2.161)	0.364		
Post-operative Radiotherapy (No vs. Yes)	1.558 (0.726–3.342)	0.255		
Tumor size (cm) (< 2 vs. 2–4 vs. ≥4)	1.528 (0. 890–2.624)	0.124		
Stromal invasion (< 1/2 vs. ≥1/2)	**2.481 (1.026–5.997)**	**0.044**	**6.377 (1.397–29.118)**	**0.017**
Endometrial invasion (No vs. Yes)	1.841 (0.711–4.769)	0.209		
Parametrium invasion (No vs. Yes)	**2.946 (1.276–6.802)**	**0.011**	**4.663 (1.496–14.533)**	**0.008**
CIN (No vs. Yes)	0.667 (0.235–1.898)	0.448		
LNM (No vs. Yes)	1.697 (0.865–3.326)	0.124		
Nerve invasion (No vs. Yes)	**3.630 (1.814–7.264)**	**0.000**	**4.044 (1.514–10.804)**	**0.005**
LVI (No vs. Yes)	2.150 (0.964–4.797)	0.061		
N-marker (≤2+ vs. > 2+)	1.777 (0.542–5.829)	0.343		
MMR (pMMR vs. dMMR)	0.039 (0.001–2.816)	0.138		
Ki67 (< 60 vs. ≥60%)	1.485 (0.522–4.223)	0.458		
SOX2 (Low vs. High)	**2.658 (1.154–6.125)**	**0.022**	**4.437 (1.276–15.428)**	**0.019**
P16^INK4A^ (− vs. +)	2.646 (0.806–8.690)	0.109		
HR-HPV RISH (− vs. +)	**3.195 (0.961–10.625)**	**0.048**	**5.160 (1.098–24.254)**	**0.038**
SOX2/ P16^INK4A^ (AI vs. AII vs. AIII)	**2.457 (1.267–4.766)**	**0.008**	1.593 (0.481–5.274)	0.446
SOX2/ HR-HPV RISH (BI vs. BII vs. BIII)	**3.462 (1.655–7.242)**	**0.001**	**3.597 (1.085–11.928)**	**0.036**

*CIN* Cervical Intraepithelial Neoplasia, *LNM* Lymph Node Metastasis, *LVI* Lymphatic Vessel Invasion, *N-marker* Neuroendocrine-marker, *MMR* Mismatch Repair, *AI* SOX2^Low^/P16^INK4A^-; AII: SOX2^High^/P16^INK4A^- or SOX2^Low^/P16^INK4A^+; AIII: SOX2^High^/P16^INK4A^+; BI: SOX2^Low^/HR-HPV RISH-; BII: SOX2^High^/HR-HPV RISH- or SOX2^Low^/HR-HPV RISH+; BIII: SOX2^High^/HR-HPV RISH+

**Table 3:** Univariate and multivariate Cox proportional hazards regression analysis for DFS

Variables	Univariate		Multivariate	
	HR (95% CI)	***P*** value	HR (95% CI)	***P*** value
Age (years) (< 44 vs. ≥44)	1.241 (0.626–2.459)	0.536		
HPV DNA (pg/ml) (< 1 vs. ≥1)	0.659 (0.087–4.978)	0.686		
FIGO stage (≤IIA vs. ≥IIB)	1.797 (0.630–5.128)	0.273		
Pathological type (SCNEC -alone vs. SCNEC -mix)	1.455 (0.685–3.092)	0.330		
Pre-operative Chemotherapy (No vs. Yes)	0.841 (0.408–1.733)	0.638		
Post-operative Chemotherapy (No vs. Yes)	0.372 (0.113–1.224)	0.104		
Post-operative Radiotherapy (No vs. Yes)	0.790 (0.398–1.565)	0.499		
Tumor size (cm) (< 2 vs. 2–4 vs. ≥4)	1.132 (0.675–1.899)	0.637		
Stromal invasion (< 1/2 vs. ≥1/2)	1.382 (0.660–2.892)	0.391		
Endometrial invasion (No vs. Yes)	1.136 (0.347–3.722)	0.833		
Parametrium invasion (No vs. Yes)	2.359 (0.910–6.119)	0.077		
CIN (No vs. Yes)	1.171 (0.509–2.696)	0.711		
LNM (No vs. Yes)	1.071 (0.536–2.141)	0.845		
Nerve invasion (No vs. Yes)	**2.707 (1.310–5.594)**	**0.007**	**3.398 (1.606–7.190)**	**0.001**
LVI (No vs. Yes)	2.061 (0.928–4.576)	0.076		
N-marker (≤2+ vs. > 2+)	1.472 (0.449–4.828)	0.523		
MMR (pMMR vs. dMMR)	0.039 (0.001–2.416)	0.123		
Ki67 (< 60 vs. ≥60%)	3.244 (0.777–13.551)	0.107		
SOX2 (Low vs. High)	**2.401 (1.044–5.524)**	**0.039**	**2.530 (1.092–5.863)**	**0.030**
P16^INK4A^ (− vs. +)	2.246 (0.685–7.366)	0.182		
HR-HPV RISH (− vs. +)	**4.309 (1.019–19.208)**	**0.047**	**6.113 (1.406–26.581)**	**0.016**
SOX2/ P16^INK4A^ (AI vs. AII vs. AIII)	**2.123 (1.108–4.067)**	**0.023**	1.239 (0.564–2.721)	0.593
SOX2/ HR-HPV RISH (BI vs. BII vs. BIII)	**3.220 (1.532–6.771)**	**0.002**	**2.880 (1.199–6.919)**	**0.018**

*CIN* Cervical Intraepithelial Neoplasia, *LNM* Lymph Node Metastasis, *LVI* Lymphatic Vessel Invasion, *N-marker* Neuroendocrine-marker, *MMR* Mismatch Repair, *AI* SOX2^Low^/P16^INK4A^-; AII: SOX2^High^/P16^INK4A^- or SOX2^Low^/P16^INK4A^+; AIII: SOX2^High^/P16^INK4A^+; BI: SOX2^Low^/HR-HPV RISH-; BII: SOX2^High^/HR-HPV RISH- or SOX2^Low^/HR-HPV RISH+; BIII: SOX2^High^/HR-HPV RISH+

### Prognostic nomograms were created to predict OS and DFS

**Fig. 6:**
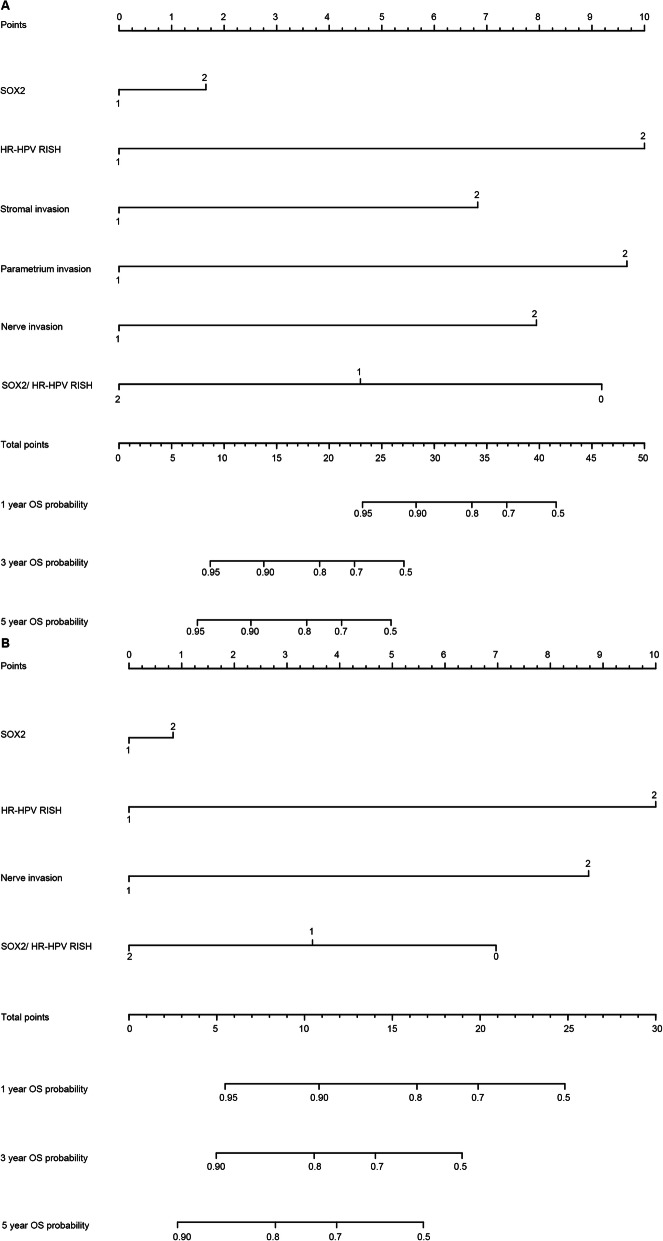
Nomograms created for predicting 1-, 3-, and 5-year OS and DFS for SCNEC. **a** Nomogram created for predicting 1-, 3-, and 5-year OS for SCNEC. **b** Nomogram created for predicting 1-, 3-, and 5-year DFS for SCNEC

**Fig. 7:**
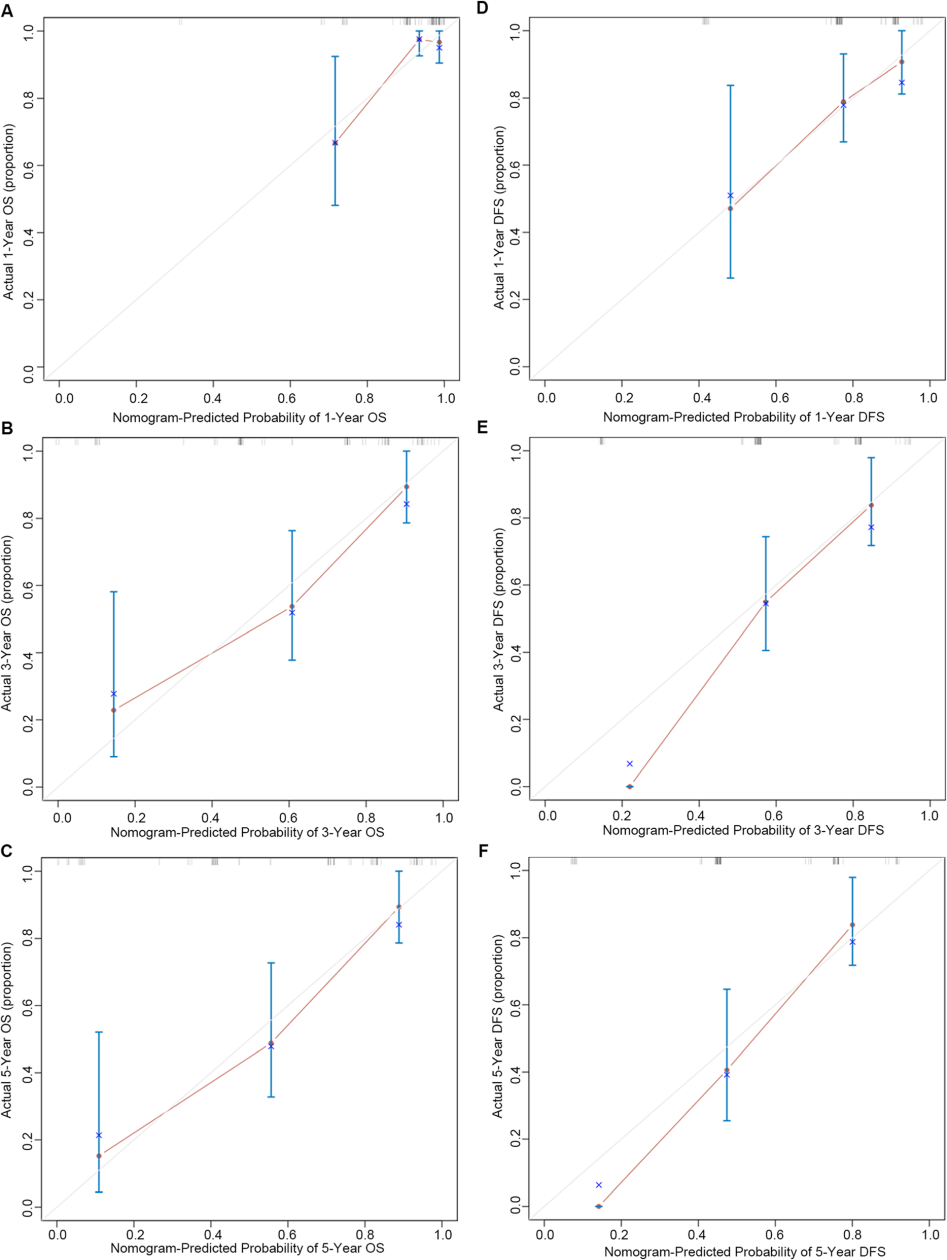
Calibration plots predicting the 1-, 3- and 5-year OS and DFS. **a**-**c** Calibration plots predicting the 1-, 3- and 5-year OS for SCNEC. (D-F) Calibration plots predicting 1-, 3- and 5-year DFS for SCNEC

Variables obtained based on Cox proportional analysis were then applied to build the respective OS and DFS prognostic nomograms (Fig. 6). The factors that were incorporated into the OS nomogram included stromal, parametrium, and nerve invasion, SOX2, HR-HPV RISH, and SOX2/HR-HPV RISH. Additionally, four risk factors—nerve invasion, SOX2, HR-HPV RISH, and SOX2/HR-HPV RISH—were enrolled in the DFS nomogram. One point was assigned to the prognostic factor in the as-constructed nomograms. Then, the total points were summed up to determine outcome probability by plotting a perpendicular line to the axis of “1-, 3-, and 5-year OS/DFS probabilities”. Figure 7 shows the calibration plots used to predict OS and DFS at 1-, 3-, and 5-year intervals, which reveal the accurate predictive power.

## Discussion

The present work examined the significance of SOX2, HR-HPV RISH, and clinicopathological features in SCNEC cases. The respective nomograms were constructed according to Cox hazards analysis to predict OS and DFS for SCNEC cases. Thereafter, each point was assessed for prognostic risk, and individualized post-treatment was provided. To our knowledge, the present work is the first retrospective analysis of the value of SOX2 and HR-HPV RISH in predicting the prognosis for SCNEC.

The dysregulated OCT4/SOX2 complex has been detected in various human malignant tumors [27], and thus, SOX2 plays a critical role in cancer development [7]. Overexpression of SOX2 has been detected in human cancers, and therefore, it may serve as an oncogene [28]. Additionally, previous studies have examined the effect of SOX2 levels on small cell neuroendocrine carcinomas (NECs) in certain organs, and found that SOX2 possibly plays a vital role in small cell NEC progression in the endometrium, esophagus, and lung [29–31]. Nonetheless, little research has focused on SOX2 expression and its clinical value in cancer. In the present study, SOX2 independently predicted the poor prognosis of SCNEC, similar to the results of prior studied [32]. Hence, SOX2 plays an important role in SCNEC development.

The role of HPV in the etiology of SCNEC is well established, and HR-HPV can be detected in the majority of the patients [33]. The presence of P16^INK4a^/Ki-67 can serve as a candidate marker for HR-HPV infection in HPV-associated endocervical neoplasia [34, 35]. However, the scoring system of 16^INK4a^ is currently controversial, often leading to a misinterpretation of the staining results [36], and the diagnostic value of Ki-67 in SCNEC remains ambiguous. HR-HPV RISH is a robust technique for HR-HPV diagnosis [37, 38] and detects the full-length or fragments of E6 and E7 transcripts using cascade signal amplification [38]. Studies have shown that persistent infection with HR-HPVs results in integration of the viral genome fragments into the host chromosomes, thus facilitating the transcription of type-specific E6/7 genes and protein overexpression, which eventually leads to the activation of the downstream carcinogenetic signaling pathways [37]. Recent studies have shown that HR-HPV RISH effectively diagnoses endocervical adenocarcinoma and endocervical glandular neoplasia [39, 40]. Therefore, a high specificity of HR-HPV RISH for HPV-driven cervical neoplasia is expected. In our study, HR-HPV RISH showed higher sensitivity and specificity for SCNEC, compared to P16^INK4a^ and Ki-67 IHC. Multivariate analysis demonstrated that SOX2/HR-HPV RISH co-expression served as an independent factor in predicting the OS and DFS in SCNEC cases. Further studies, using larger cohorts, should be conducted to validate our findings.

Consistent with our results, SOX2 was proven to be a potential marker to predict overall survival and recurrence in p16+ oropharyngeal cancer [41]. Recent studies have shown that SOX2 was related to HPV infection. Interestingly, HPV infection drives switches in SOX2 expression in the transformation zone in the uterine cervix [15], and SOX2 locus amplification was related with HPV mRNA positivity in vulvar carcinoma [14]. Furthermore, SOX2 was reported to be regulator of HPV16 at the transcriptional level in cervical squamous cell carcinoma [42]. That may explain the possible molecular mechanism between them. In our study, both SOX2 and HR-HPV RISH were independent prognostic factors for SCNEC. Unfortunately, there is no significant difference between the expression of SOX2 and HR-HPV RISH in the correlation analysis, which may also be related to the sample size. Therefore, the possible molecular mechanism between SOX2 and HPV infection in SCNEC remains to be further studied.

There were several limitations to this study. First, its retrospective nature may lead to inevitable selection bias. Second, this study was conducted with a small sample size from a single center. Third, the present work only focused on the significance of SOX2 and HR-HPV RISH in predicting prognosis, while other prognostic factors, such as molecular biomarkers or inflammatory prognostic markers, were not included. Therefore, the results of this work should be further validated in multi-center studies with a larger sample size.

## Supplementary Information

**Additional file 1.**

## Data Availability

All data generated or analyzed during this study are included in this article [and its supplementary information files].
