# Correction to: Co-expression of SOX2 and HR-HPV RISH predicts poor prognosis in small cell neuroendocrine carcinoma of the uterine cervix

**DOI:** 10.1186/s12885-021-08159-y

**Published:** 2021-04-15

**Authors:** Shi-Wen Zhang, Rong-Zhen Luo, Xiao-Ying Sun, Xia Yang, Hai-Xia Yang, Si-Ping Xiong, Li-Li Liu

**Affiliations:** 1 Department of Pathology, Sun Yat-sen University Cancer Center, Guangzhou, 510060 China; 2 Department of Pathology, the Eighth Affiliated Hospital, Sun Yat-sen University, Shenzhen, 51800 China; 3 Department of Gynecological Oncology, Sun Yat-sen University Cancer Center, Guangzhou, 510060 China

**Correction to: BMC Cancer 21, 332 (2021)**

**https://doi.org/10.1186/s12885-021-08059-1**

Following publication of the original article [1], the authors reported an error in the order of the affiliations. The correct affiliations are reflected in this article. The original article [1] has been corrected.
