# Supplementary material for: Co-expression of SOX2 and HR-HPV RISH predicts poor prognosis in small cell neuroendocrine carcinoma of the uterine cervix
Source: BMC Cancer. 2021 Mar 31;21:332. doi: 10.1186/s12885-021-08059-1 (PMC8011148; doi:10.1186/s12885-021-08059-1)
Supplement: Supplementary file 1 — Additional file 1. [file 12885_2021_8059_MOESM1_ESM.pdf]

Co-expression of SOX2 and HR-HPV RISH predicts poor prognosis in small cell neuroendocrine carcinoma of the uterine cervix

Shi-Wen Zhang[1,2,3#], Rong-Zhen Luo[1,2#], Xiao-Ying Sun[1,4#], Xia Yang[1,2], Hai-Xia Yang[1,2], Si-Ping Xiong[1,2], Li-Li Liu[1,2*]

Supplementary Table 1. Summary of patient characteristics.

Characteristic	Number of patients (%)
**Total**	88 (100.0)
**Age range (years)**	24-66
**Median age (years)**	44
<44	40 (45.5)
≥44	48 (54.5)
**FIGO stage**	
≤IIA	79 (89.8)
≥IIB	9 (10.2)
**Preoperative Chemotherapy**	
NO	54 (62.8)
YES	32 (37.2)
**Postoperative chemotherapy**	
NO	4 (4.5)
YES	84 (95.5)
**Postoperative radiotherapy**	
NO	33 (37.5)
YES	55 (62.5)
**Tumor size (cm)**	
<2	11 (12.5)
2-4	40 (45.5)
≥4	37 (42.0)
**Stromal invasion**	
<1/2	28 (31.8)
≥1/2	60 (68.2)
**Endometrial invasion**	
NO	79 (89.2)
YES	9 (10.8)
**Parametrium invasion**	
NO	78 (88.6)
YES	10 (11.4)
**CIN**	
NO	73 (83.0)
YES	15 (17.0)

**LNM**	
NO	52 (59.1)
YES	36 (40.9)
**LVI**	
NO	29 (33.0)
YES	59 (67.0)
**Nerve invasion**	
NO	67 (76.1)
YES	21 (23.9)
**$P16^{INK4A}$**	
Negative	12 (13.6)
Positive	76 (86.4)
**MMR**	
pMMR	78 (88.6)
dMMR	10 (11.4)
**Pathological type**	
SCNEC -alone	66 (75.0)
SCNEC -mix	22 (25.0)
**SOX2**	
Negative	20 (22.7)
Positive	68 (77.3)
**HR-HPV RISH**	
Negative	15 (17.0)
Positive	73 (83.0)

CIN：Cervical Intraepithelial Neoplasia；LNM：Lymph Node Metastasis；LVI：Lymphatic Vessel Invasion；N-marker：Neuroendocrine-marker；MMR：Mismatch Repair

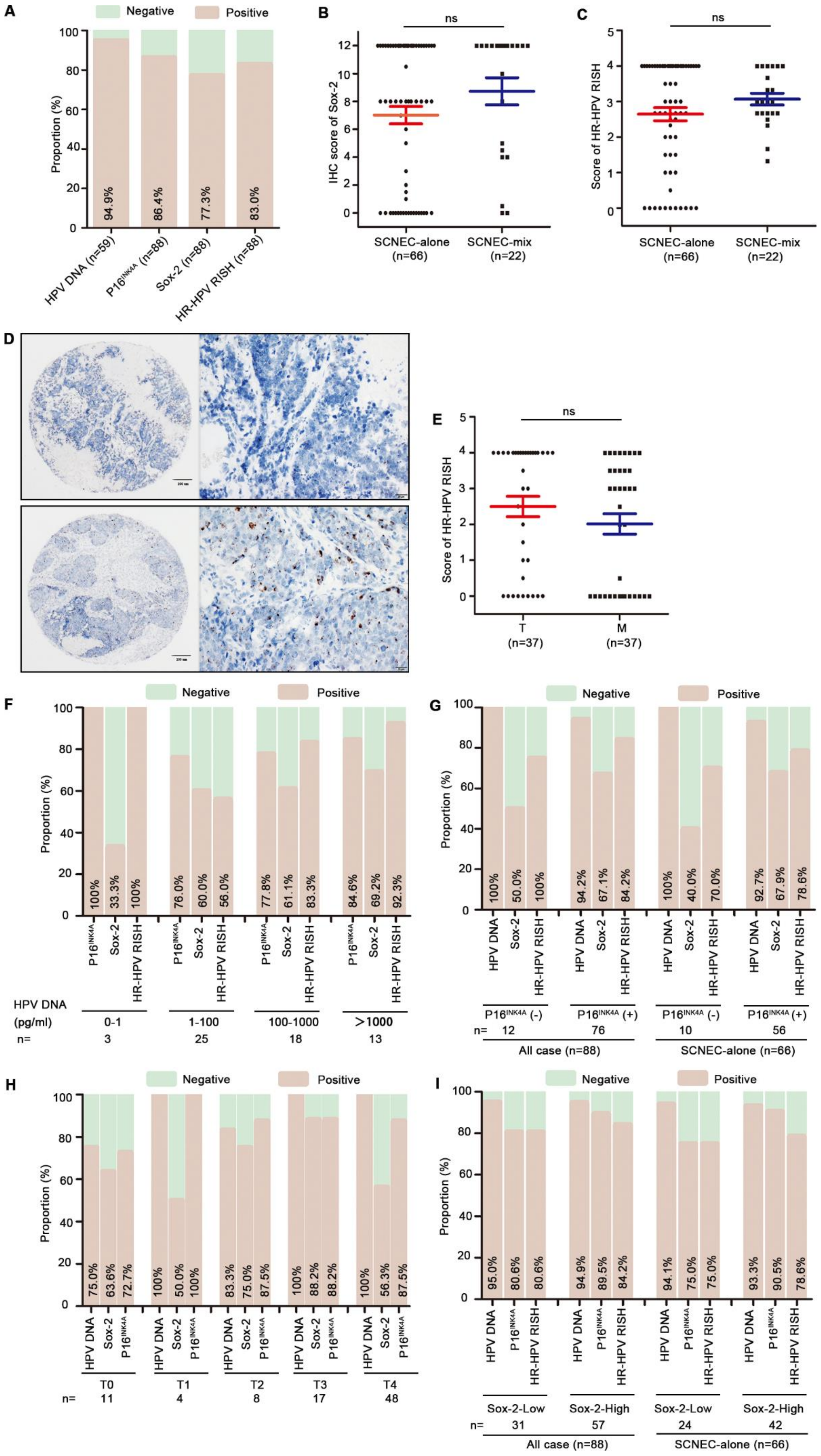
A
Negative
Positive
Proportion (%)
94.9%
86.4%
77.3%
83.0%
HPV DNA (n=59)
P16INK4A (n=88)
Sox-2 (n=88)
HR-HPV RISH (n=88)
B
ns
IHC score of Sox-2
SCNEC-alone (n=66)
SCNEC-mix (n=22)
C
ns
Score of HR-HPV RISH
SCNEC-alone (n=66)
SCNEC-mix (n=22)
D
E
ns
Score of HR-HPV RISH
T (n=37)
M (n=37)
F
Negative
Positive
Proportion (%)
100%
33.3%
100%
76.0%
60.0%
56.0%
77.8%
61.1%
83.3%
84.6%
69.2%
92.3%
P16INK4A
Sox-2
HR-HPV RISH
HPV DNA (pg/ml)
0-1
1-100
100-1000
>1000
n=
3
25
18
13
G
Negative
Positive
Proportion (%)
100%
50.0%
100%
94.2%
67.1%
84.2%
100%
40.0%
70.0%
92.7%
67.9%
78.6%
HPV DNA
Sox-2
HR-HPV RISH
P16INK4A (-)
P16INK4A (+)
n=
12
76
10
56
All case (n=88)
SCNEC-alone (n=66)
H
Negative
Positive
Proportion (%)
75.0%
63.6%
72.7%
100%
50.0%
100%
83.3%
75.0%
87.5%
100%
88.2%
88.2%
100%
56.3%
87.5%
HPV DNA
Sox-2
P16INK4A
T0
T1
T2
T3
T4
n=
11
4
8
17
48
I
Negative
Positive
Proportion (%)
95.0%
80.6%
80.6%
94.9%
89.5%
84.2%
94.1%
75.0%
75.0%
93.3%
90.5%
78.6%
HPV DNA
P16INK4A
HR-HPV RISH
Sox-2-Low
Sox-2-High
n=
31
57
24
42
All case (n=88)
SCNEC-alone (n=66)

Supplementary Figure 1

Supplementary Figure 1. The positive rates of HPV DNA, P16^{INK4A}, SOX2, and HR-HPV RISH in the subgroups of SCNEC cases. (A) The positive proportion of HPV DNA, P16^{INK4A}, SOX2, HR-HPV RISH examined in the SCNEC samples. (B) Immunohistochemistry (IHC) scores of TMA and comparison of the SOX2 levels between SCNEC-alone and SCNEC-mix tissues. Quantitative results expressed as mean $\pm$ standard deviation (SD). (C) Comparison of the HR-HPV RISH levels between the SCNEC-alone and SCNEC-mix tissues. (D) HPV mRNA was detected using HR-HPV RISH in 37 SCNEC patients with lymph node metastases. Typical images of primary tumor (T) and metastasis (M). (E) HR-HPV RISH expression in the primary tumor (T) and metastasis (M). (F-I) The positive rates of P16^{INK4A}, SOX2, and HR-HPV RISH expression in the subgroups of SCNEC cases.

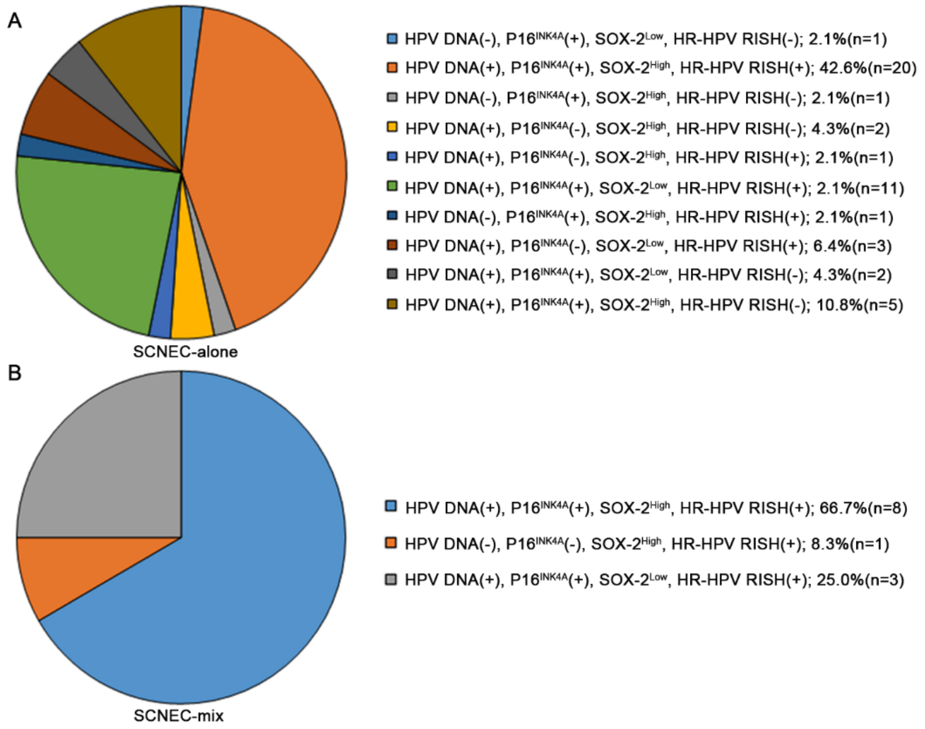

Supplementary Figure 2

Supplementary Figure 2. SCNEC: proportions of the combinations of positive HPV tests, P16^{INK4A}, and SOX2 IHC. Proportions of the combinations of positive HPV tests, P16^{INK4A}, and SOX2 IHC in SCNEC-alone (A) and SCNEC-mix tissues (B).

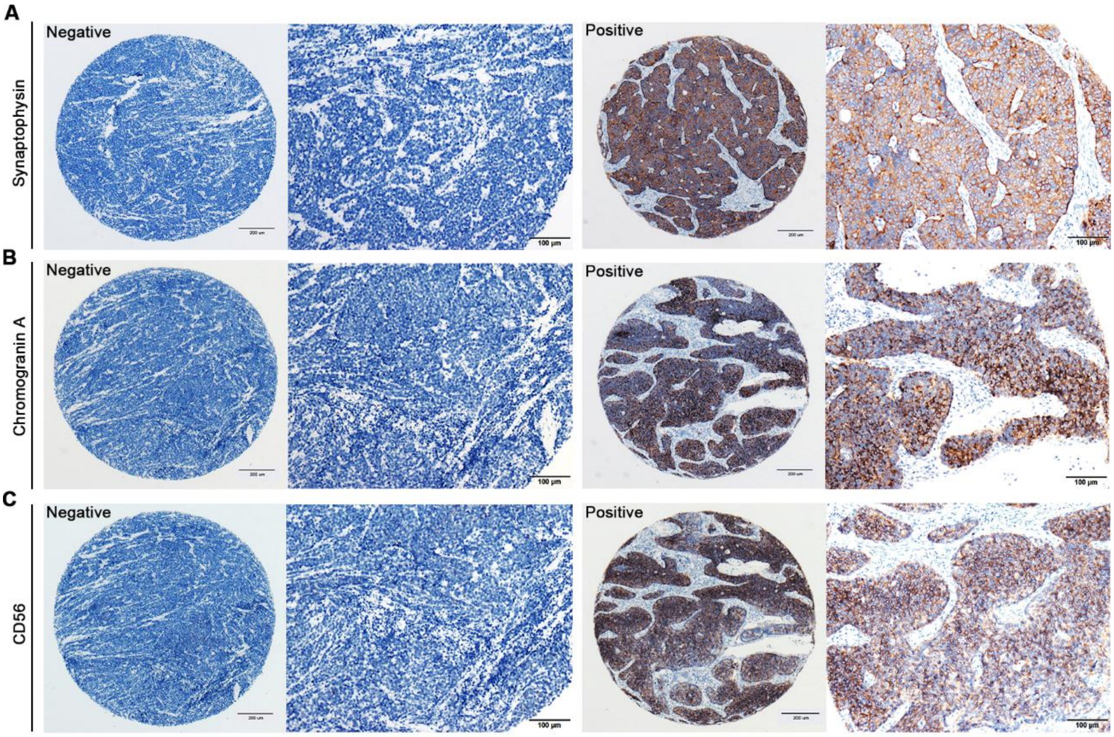

Supplementary Figure 3

Supplementary Figure 3. Negative and positive immunohistochemical staining for Synaptophysin expression (A)、chromogranin A expression (B) and CD56 expression (C) in SCNEC tumor tissues.
